# Supplementary material for: Anticipation of sexually arousing visual event leads to overestimation of elapsed time
Source: PLoS One. 2024 Jul 12;19(7):e0295216. doi: 10.1371/journal.pone.0295216 (PMC11244774; doi:10.1371/journal.pone.0295216)
Supplement: S1 Appendix — (DOCX) [file pone.0295216.s001.docx]

# Supporting Information

## S1 Appendix

**IAPS pictures:**

Pictures of couples:

4608, 4611, 4650, 4651, 4652, 4653, 4656, 4658, 4659, 4660, 4664,4 666, 4668, 4669, 4670, 4672, 4677, 4680, 4681, 4690, 4694, 4695, 4697, 4698, 4800, 4810

Pictures of women:

4085, 4090, 4130, 4141, 4142, 4180, 4210, 4220, 4225, 4232, 4235, 4240, 4250, 4255, 4290, 4300, 4302, 4310, 4311, 4320;

Pictures of men:

4460, 4470, 4490, 4500, 4503, 4505, 4510, 4520, 4525, 4530, 4531, 4533, 4538, 4542, 4550, 4559, 4561, 4572, 4574, 4575
