# Supplementary material for: Anticipation of sexually arousing visual event leads to overestimation of elapsed time
Source: PLoS One. 2024 Jul 12;19(7):e0295216. doi: 10.1371/journal.pone.0295216 (PMC11244774; doi:10.1371/journal.pone.0295216)
Supplement: Attachment — Submitted filename: Rebuttal_letter.docx [file pone.0295216.s003.docx]

# Rebuttal letter

First, we would like to thank the reviewers and the editor for their thorough work, constructive comments, and suggestions. In response to the previous comments, we have incorporated new literature to the discussion and checked the references for retracted papers or papers that have been cited in the text but were not listed in the References section.

In the following, we the remaining questions and comments.

Journal Requirements:

Please review your reference list to ensure that it is complete and correct. If you have cited papers that have been retracted, please include the rationale for doing so in the manuscript text, or remove these references and replace them with relevant current references. Any changes to the reference list should be mentioned in the rebuttal letter that accompanies your revised manuscript. If you need to cite a retracted article, indicate the article’s retracted status in the References list and also include a citation and full reference for the retraction notice.

Response: We have now gone through the references for missing or rectracted papers. No retracted papers were found. There were only two papers: one with erratum (error in figure labels) and one with a commentary. The references were formatted to PloS one- style and numbered citations were used in the text.

Additional Editor Comments:

1) it would be useful to add a sentence in the Discussion, mentioning the important role of anticipation of salient events in sexually dysfunctional patients. (e.g., citing the reference: Altered reward processing in patients with lifelong premature ejaculation. Scientific Reports.)

Response: Thank you for the suggestion. We have now added the following passage to the discussion section to take into account the relevance of our findings for sexual dysfunctions.

“Previous research on sexual dysfunctions, such as premature ejaculation (PE) disorder, has shown that men with inability to delay ejaculation during intercourses how also faster reactions when anticipating erotic images and reduced hedonic valuation of non-sexual rewards [48]. The current findings imply that the greater hedonic valuation of sexual rewards results also to stronger temporal dilation. Whether the temporal distortions caused by anticipated sexual rewards are stronger in people with sexual dysfunctions and addictions should be investigated in the future.”

2) Please choose a public repository to host your data, such as the Open Science Framework (https://osf.io) or Zenodo (https://zenodo.org). This way, the data remains accessible to interested researchers.

Response: the data has been openly available at Open Science framework. The link to the repository is provided in the data availability statement.

As there were no further comments from the reviewers, we would like to thank the Editor and the reviewers for their work on this manuscript that greatly improved in the process. We hope that the manuscript now meets the PLOS ONE’s publication criteria.
